# Supplementary figures and images for: Exploring the occurrence of thioflavin-T-positive insulin amyloid aggregation intermediates
Source: PeerJ. 2021 Feb 10;9:e10918. doi: 10.7717/peerj.10918 (PMC7881721; doi:10.7717/peerj.10918)

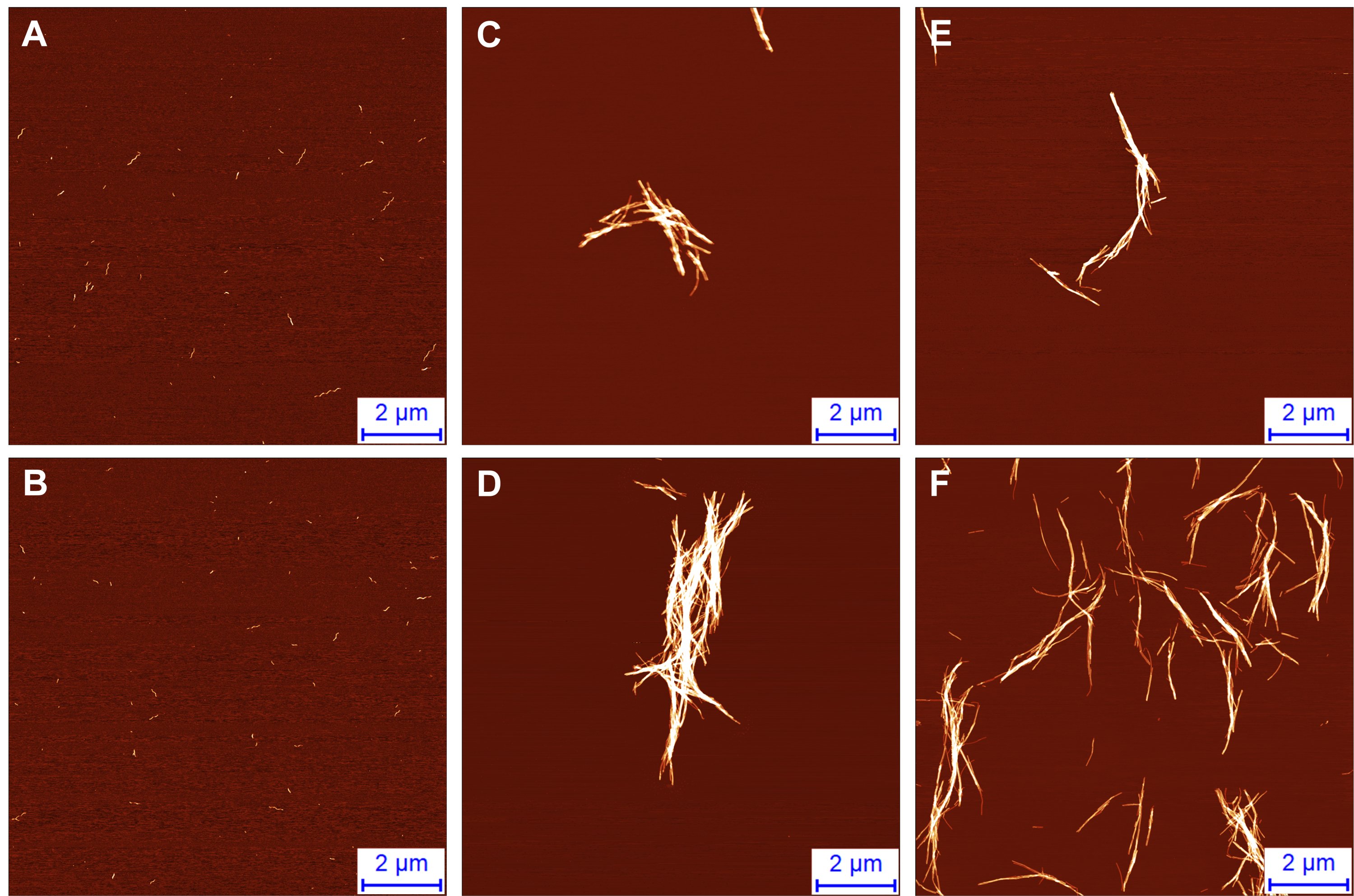

Supplement: Figure S1 [file peerj-09-10918-s001.png]

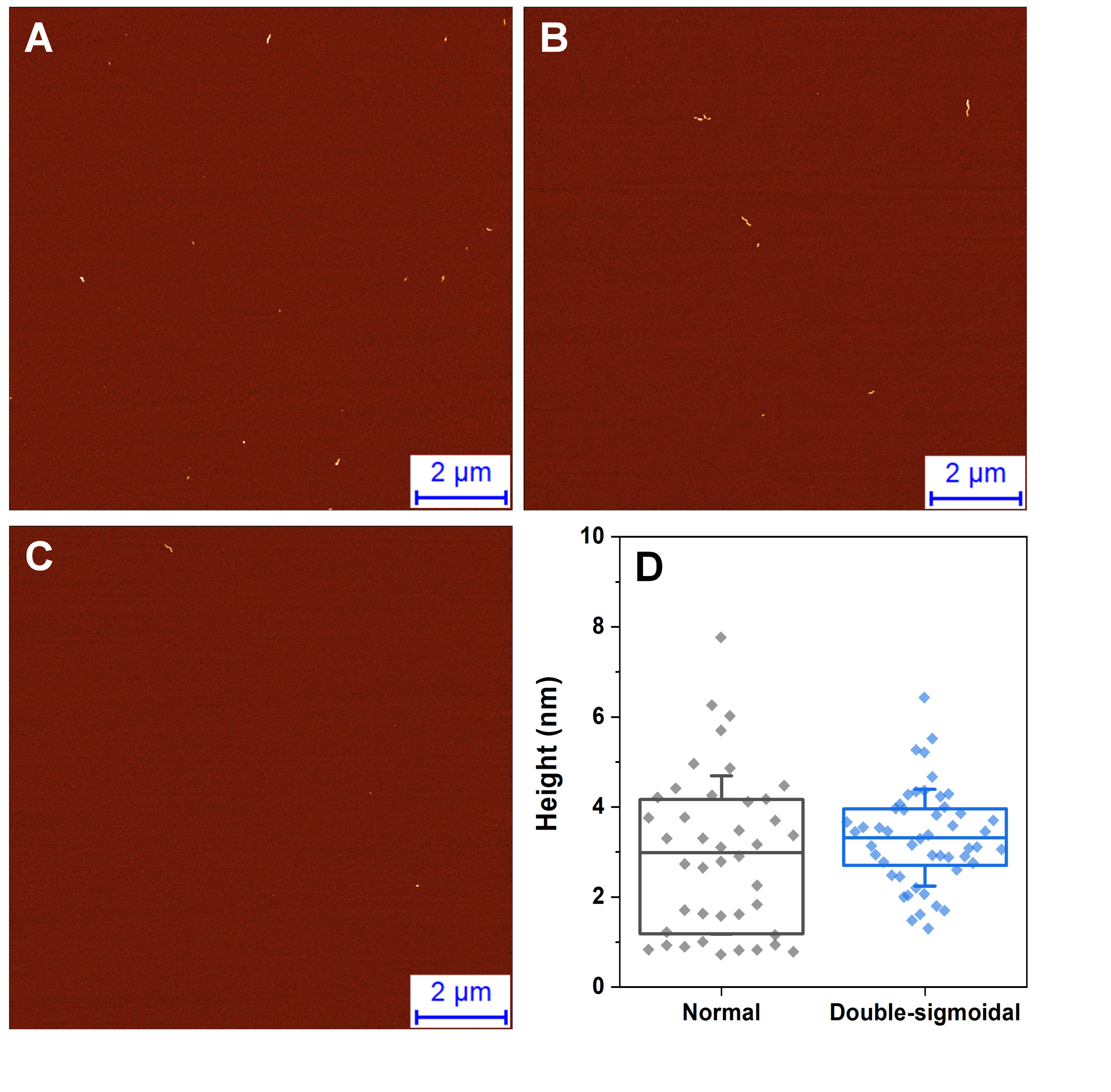

Supplement: Figure S2 — Aggregate height distribution comparison between intermediates formed during normal and double-sigmoidal aggregation (D). Intermediate aggregates were collected and deposited on freshly cleaved mica before an increase in ThT fluorescence intensity was observed. [file peerj-09-10918-s002.png]

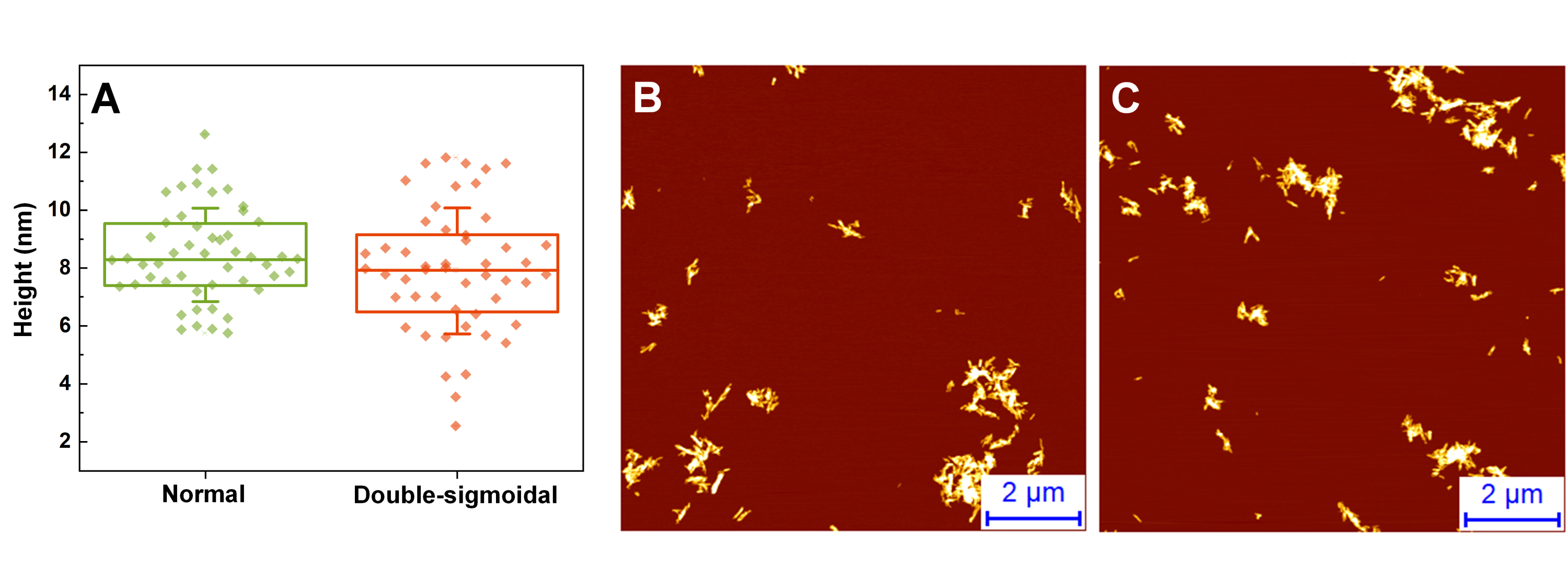

Supplement: Figure S3 — AFM images were acquired after samples were sonicated as described in the ‘Materials and Methods’ section. [file peerj-09-10918-s003.png]

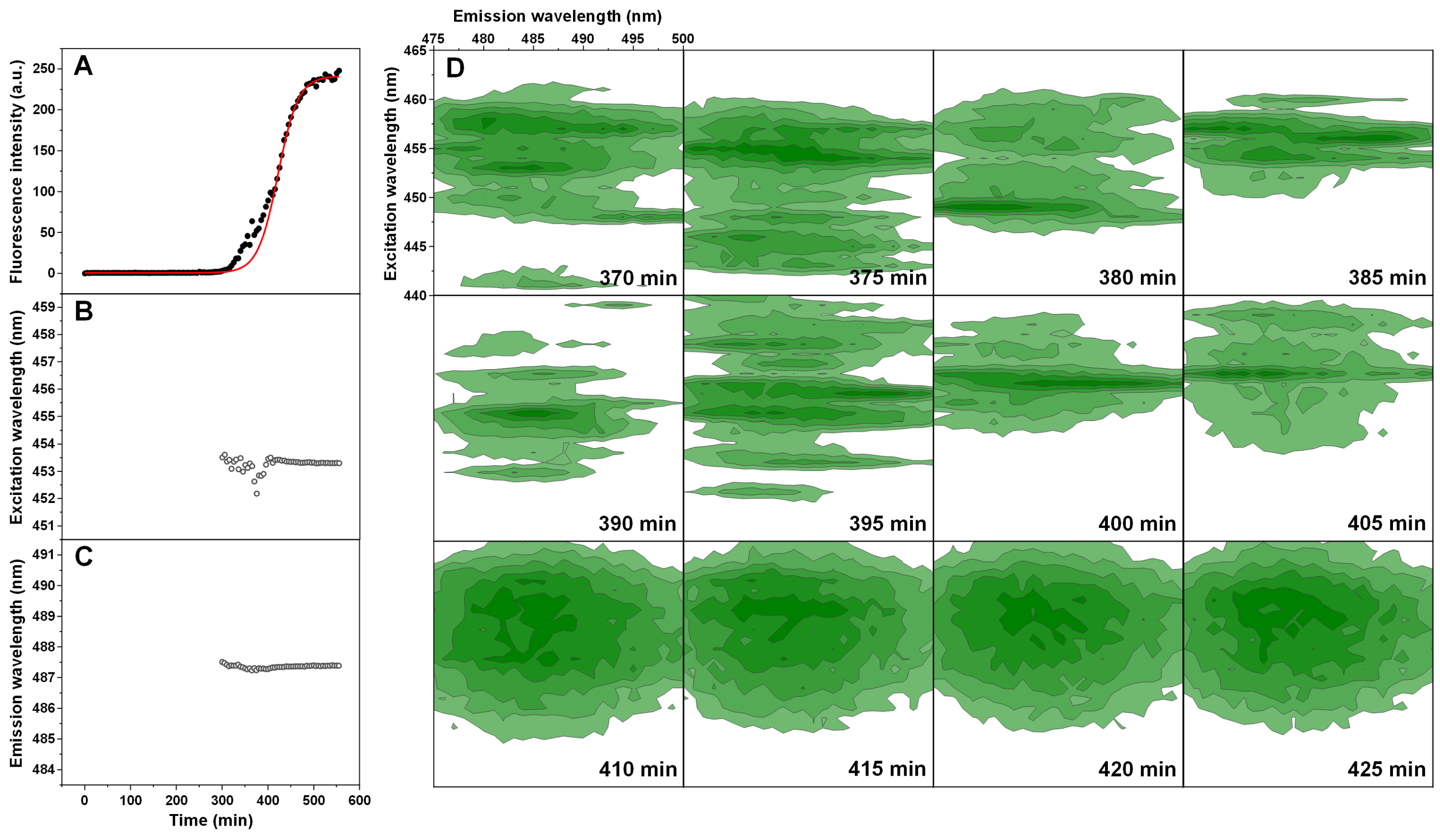

Supplement: Figure S4 — Insulin aggregation kinetics monitored by scanning ThT fluorescence EEMs (A), where each data point is the maximum value in the recorded EEM. EEM “center of mass” excitation (B) and emission (C) wavelengths over the course of aggregation. Top intensity values present in the ThT EEMs at different aggregation time points (D) during the first double-sigmoidal increase (darker green areas represent higher intensity zones). Data in part (A) was fit using a Boltzmann’s sigmoidal equation with the anomalous aggregation phase data points omitted from the fitting procedure. [file peerj-09-10918-s004.png]

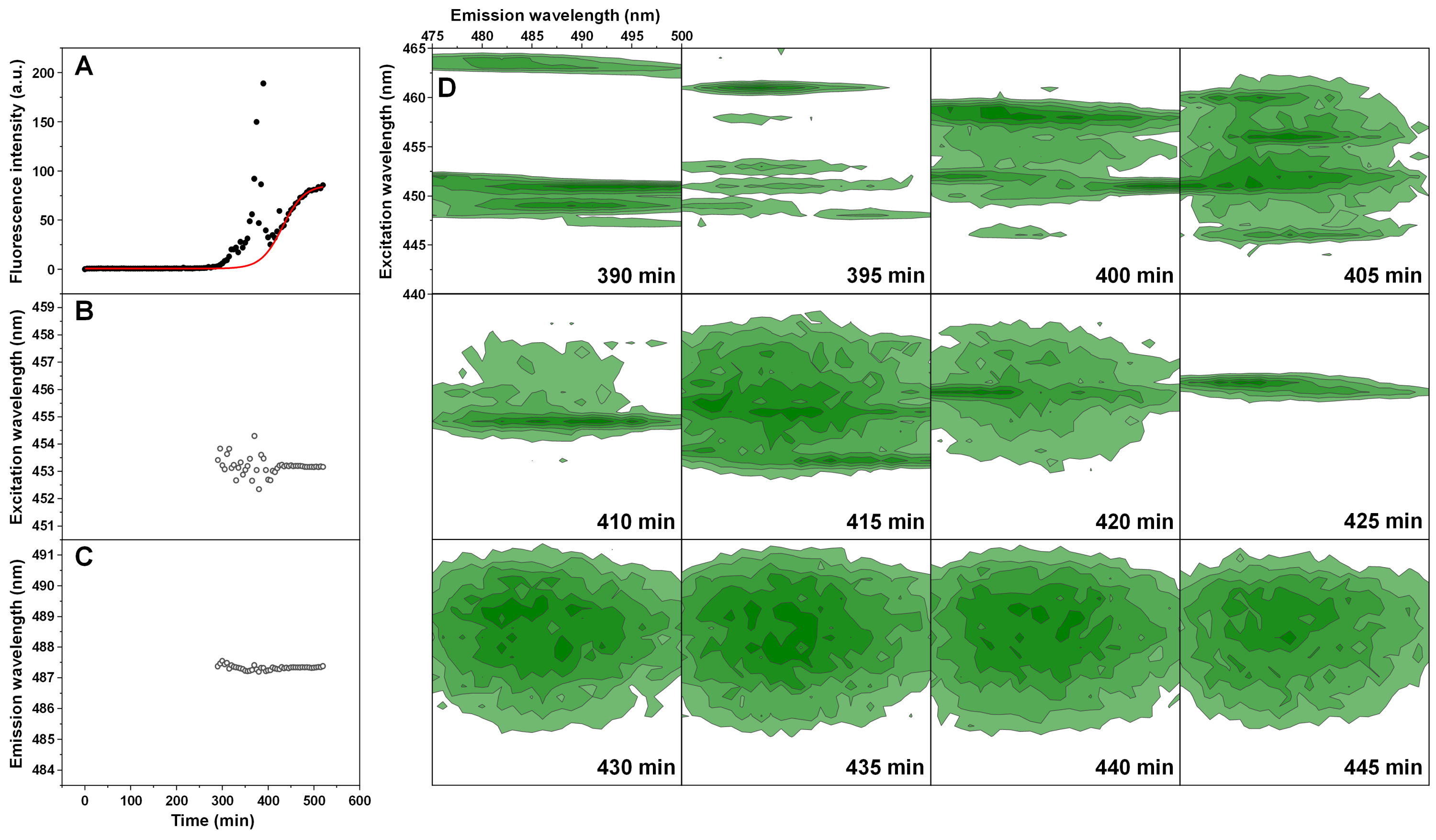

Supplement: Figure S5 — Insulin aggregation kinetics monitored by scanning ThT fluorescence EEMs (A), where each data point is the maximum value in the recorded EEM. EEM “center of mass” excitation (B) and emission (C) wavelengths over the course of aggregation. Top intensity values present in the ThT EEMs at different aggregation time points (D) during the first double-sigmoidal increase (darker green areas represent higher intensity zones). Data in part (A) was fit using a Boltzmann’s sigmoidal equation with the anomalous aggregation phase data points omitted from the fitting procedure. [file peerj-09-10918-s005.png]

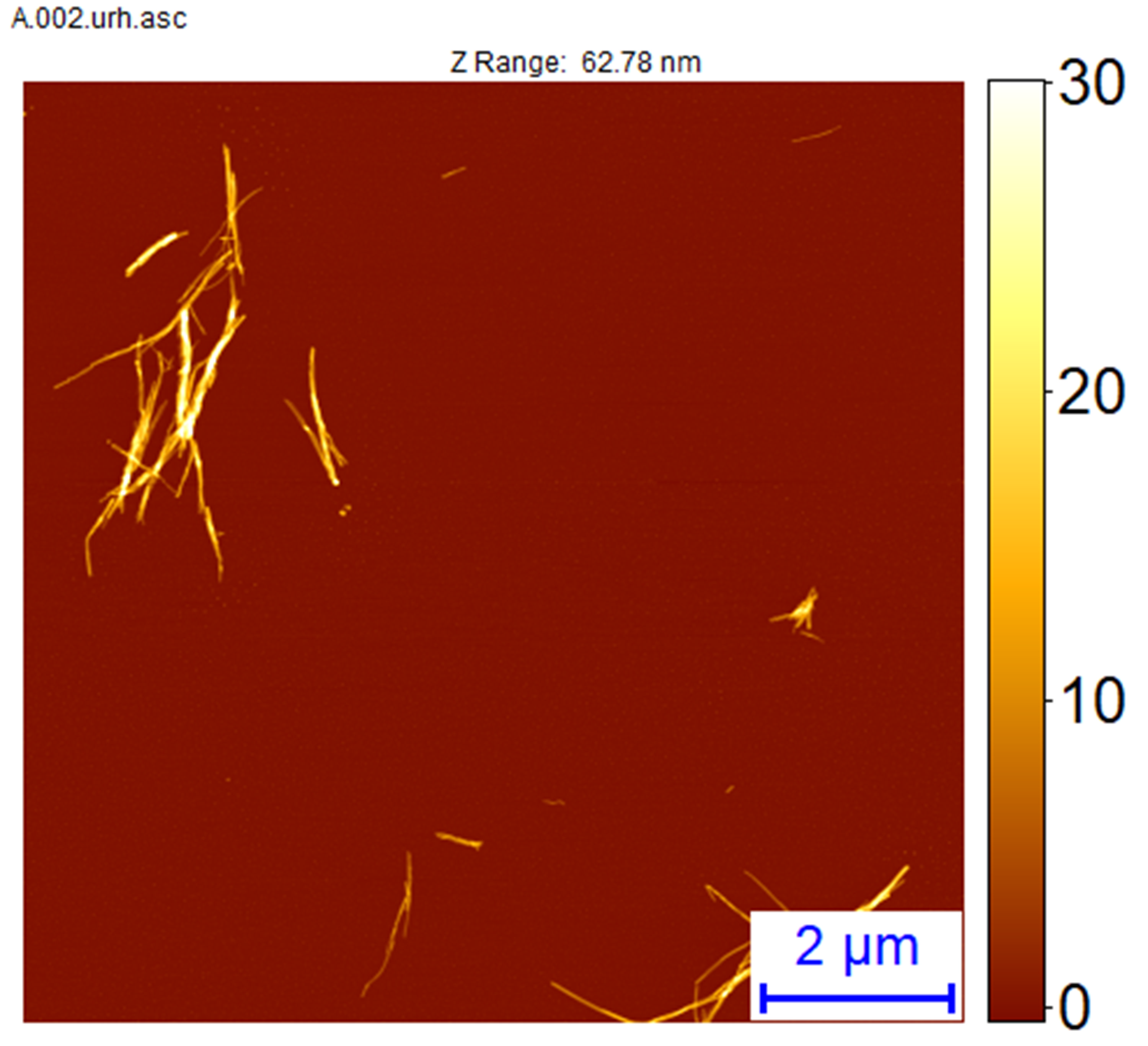

Supplement: Supplemental Information 5 [file peerj-09-10918-s010.zip › DoubleSigmoidal1.tif]

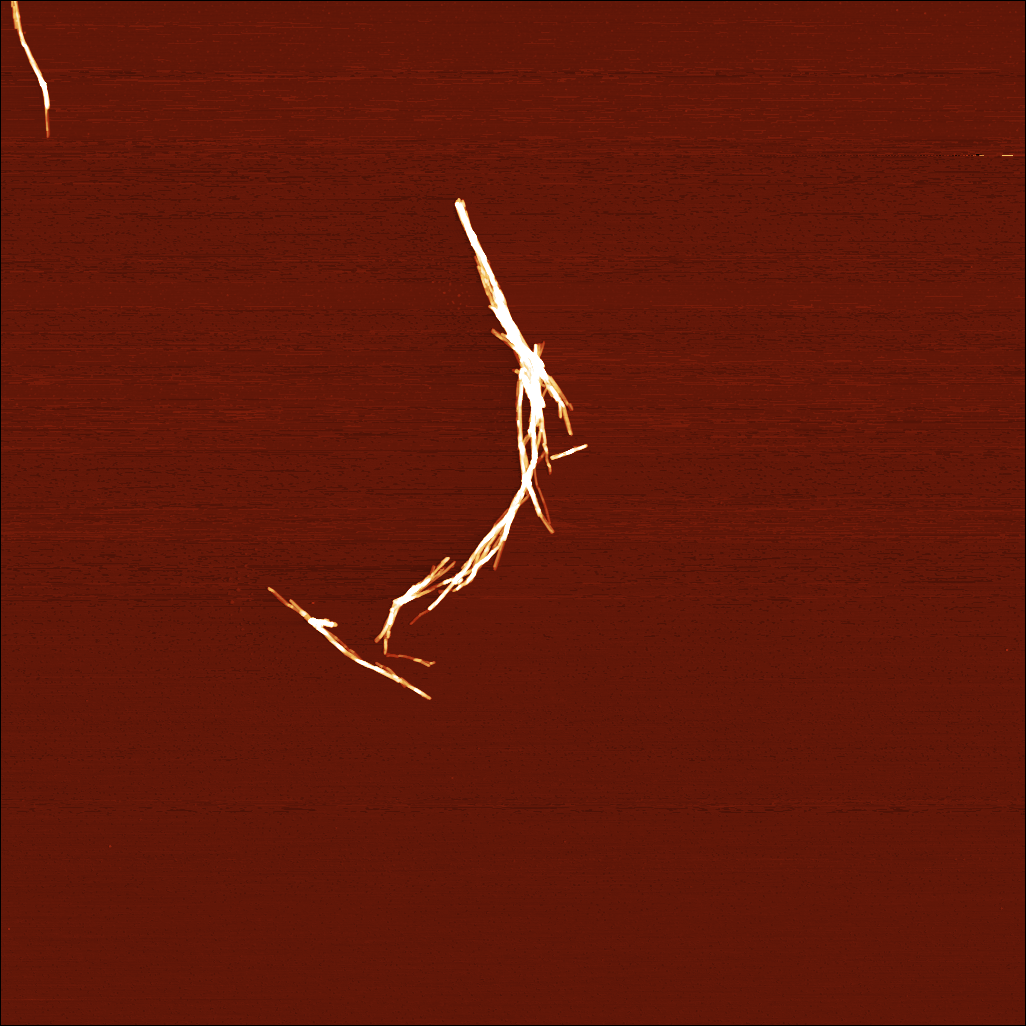

Supplement: Supplemental Information 5 [file peerj-09-10918-s010.zip › DoubleSigmoidal2.tiff]

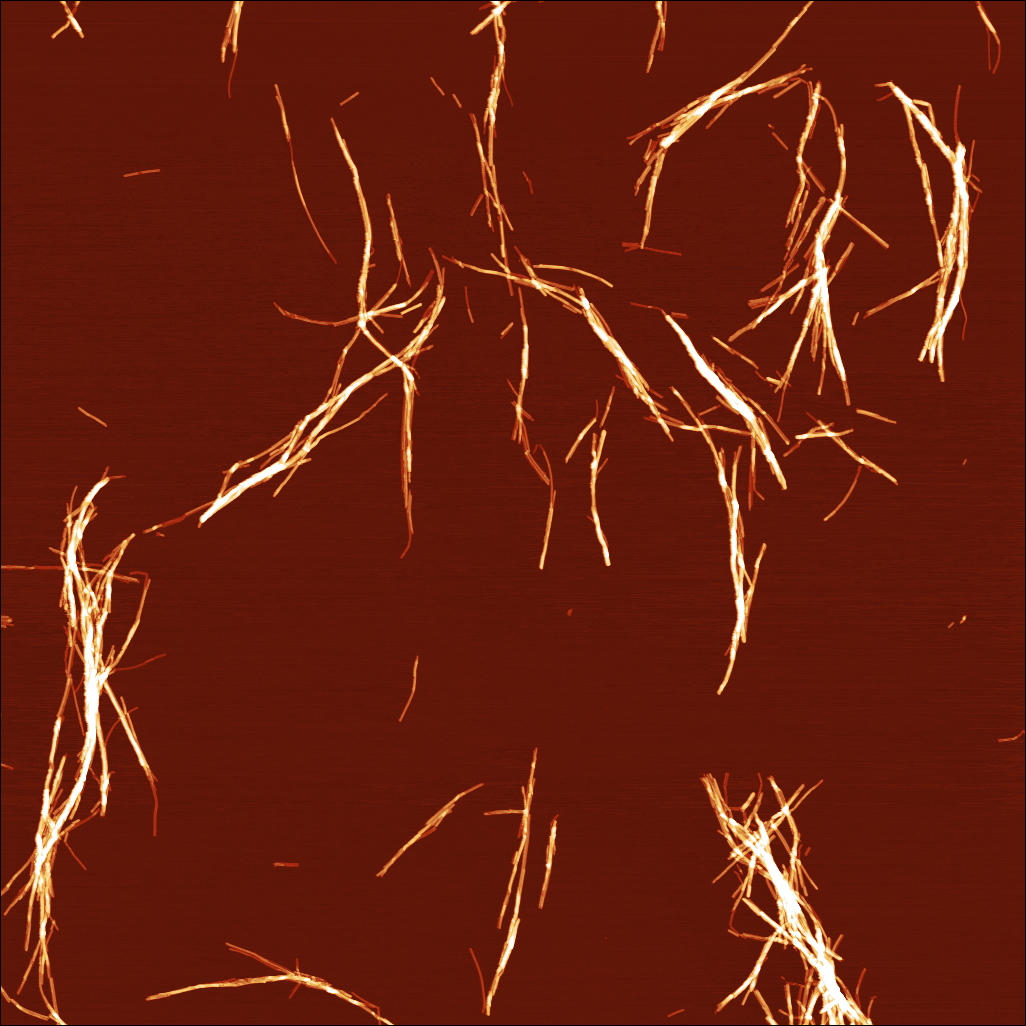

Supplement: Supplemental Information 5 [file peerj-09-10918-s010.zip › DoubleSigmoidal3.tiff]

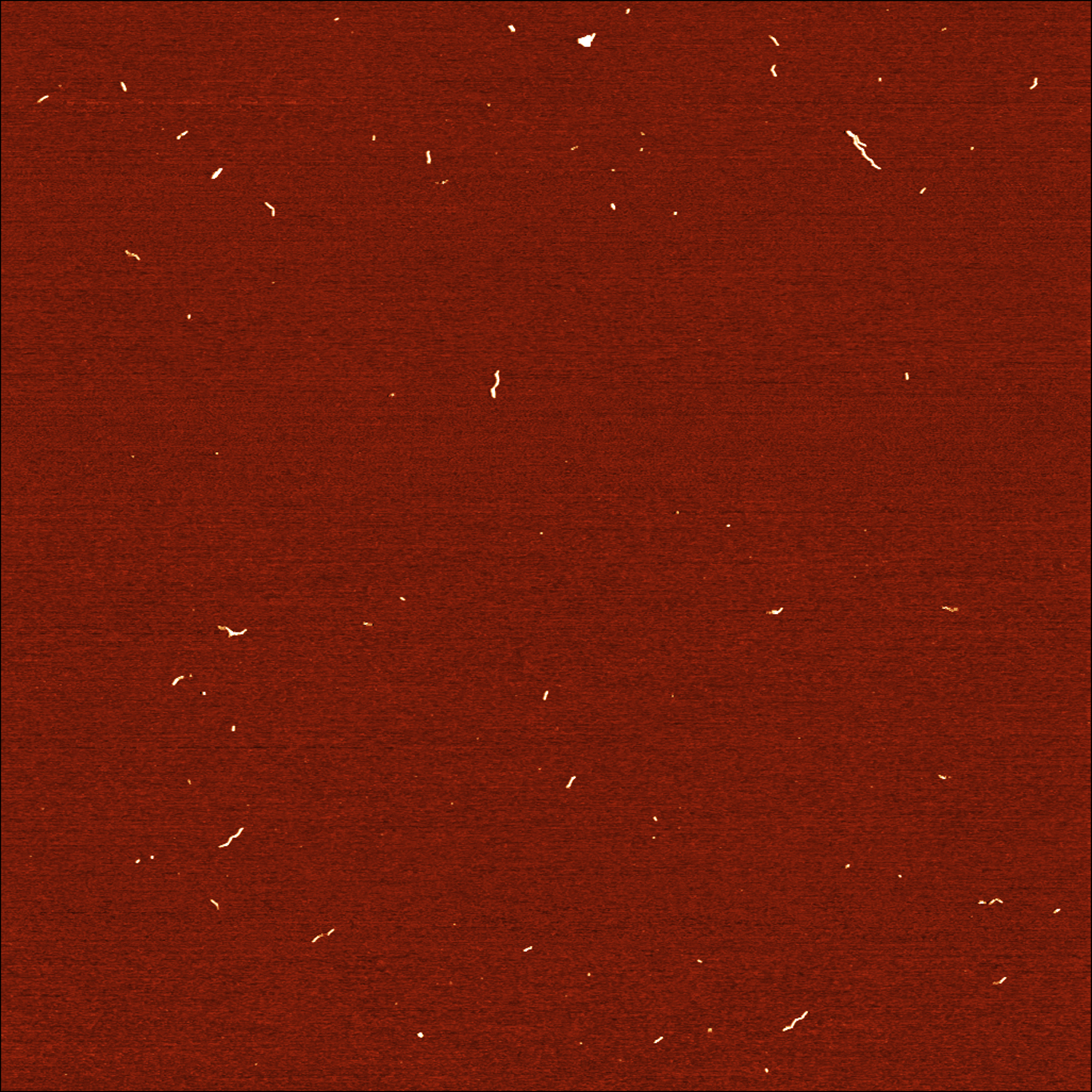

Supplement: Supplemental Information 5 [file peerj-09-10918-s010.zip › DoubleSigmoidalIntermediate1.tif]

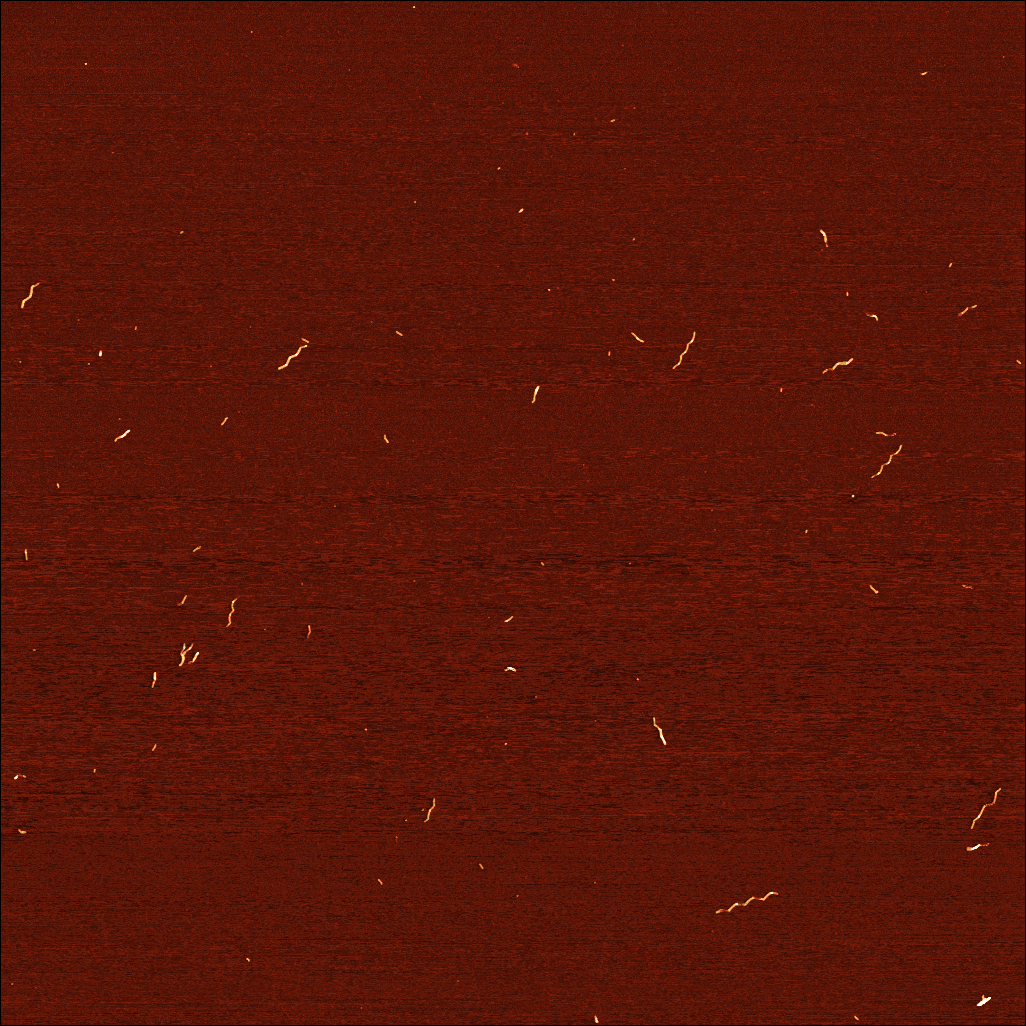

Supplement: Supplemental Information 5 [file peerj-09-10918-s010.zip › DoubleSigmoidalIntermediate2.tiff]

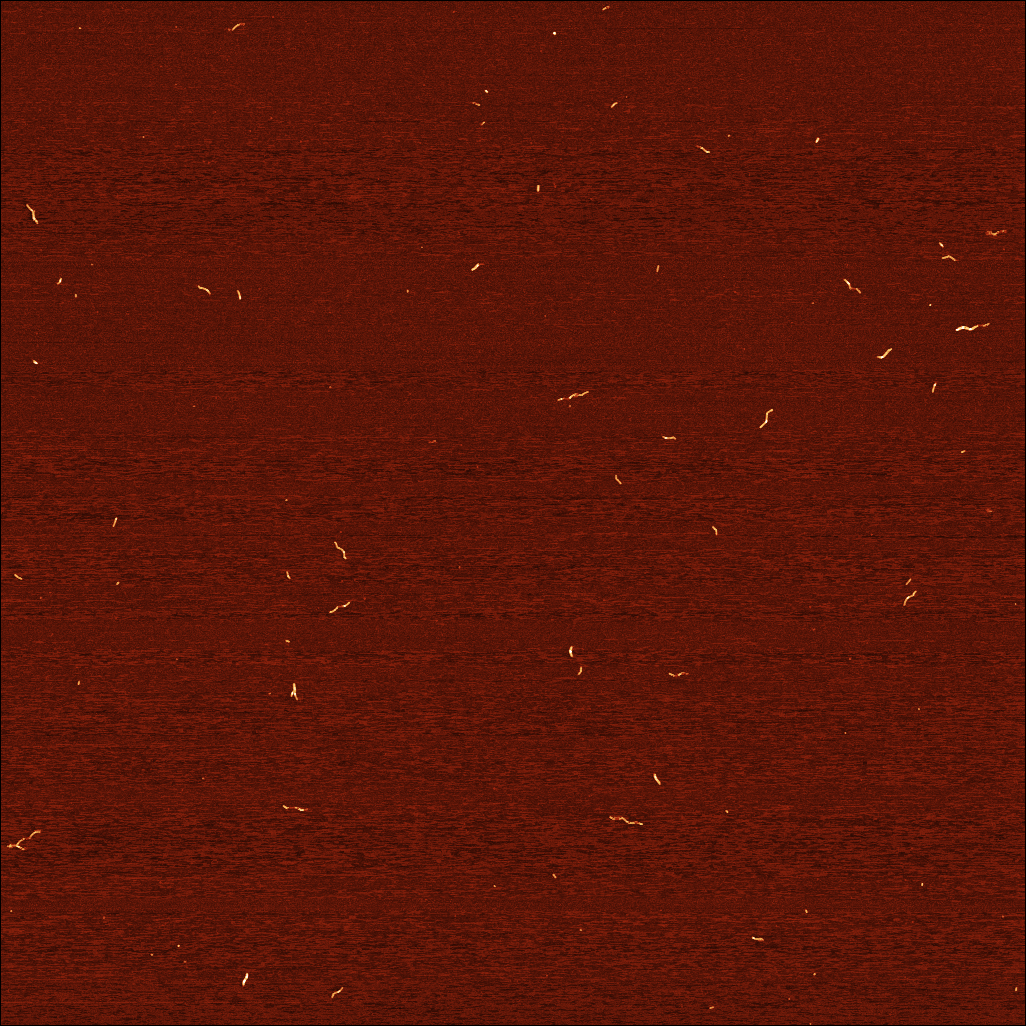

Supplement: Supplemental Information 5 [file peerj-09-10918-s010.zip › DoubleSigmoidalIntermediate3.tiff]

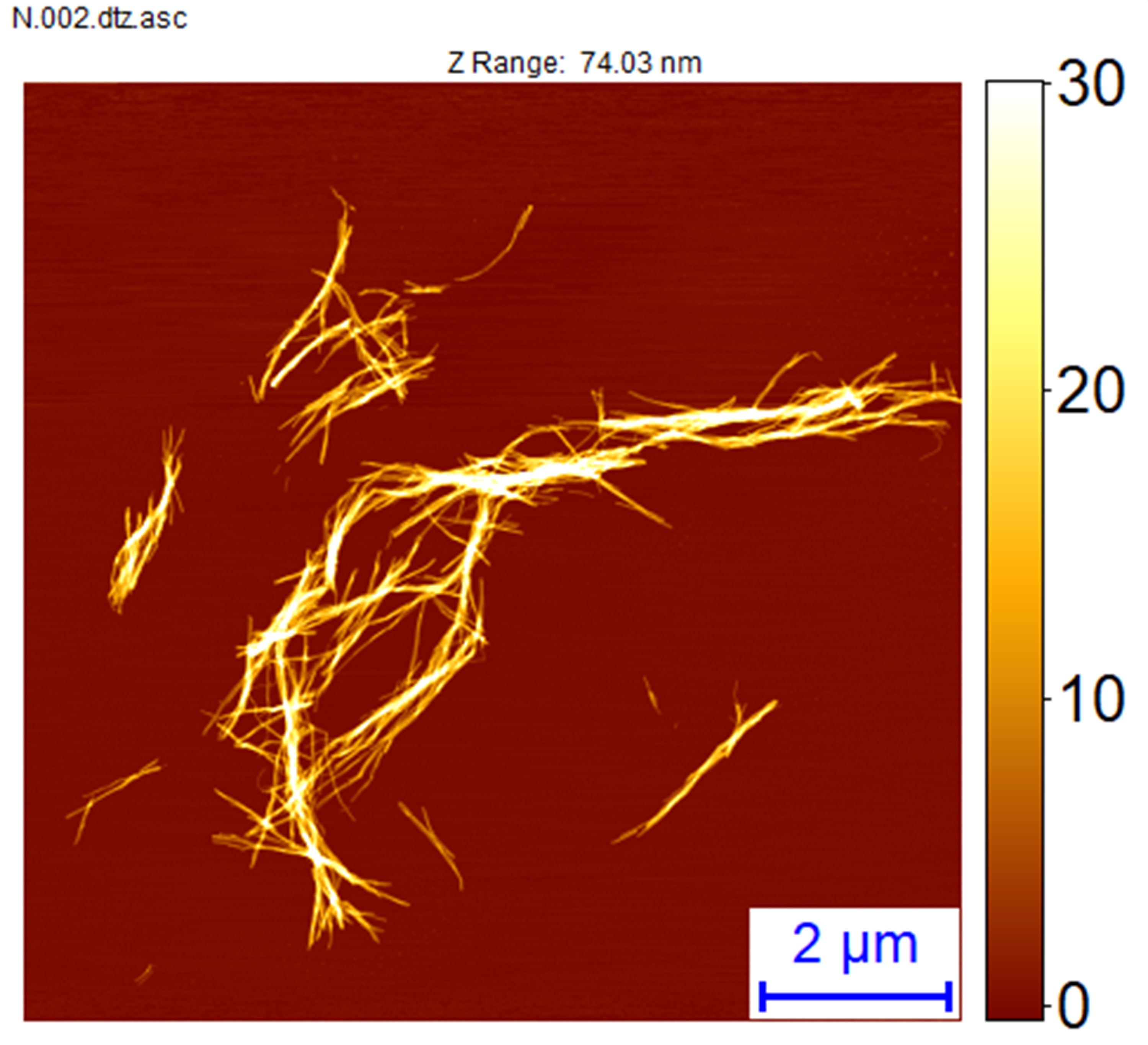

Supplement: Supplemental Information 5 [file peerj-09-10918-s010.zip › Normal1.tif]

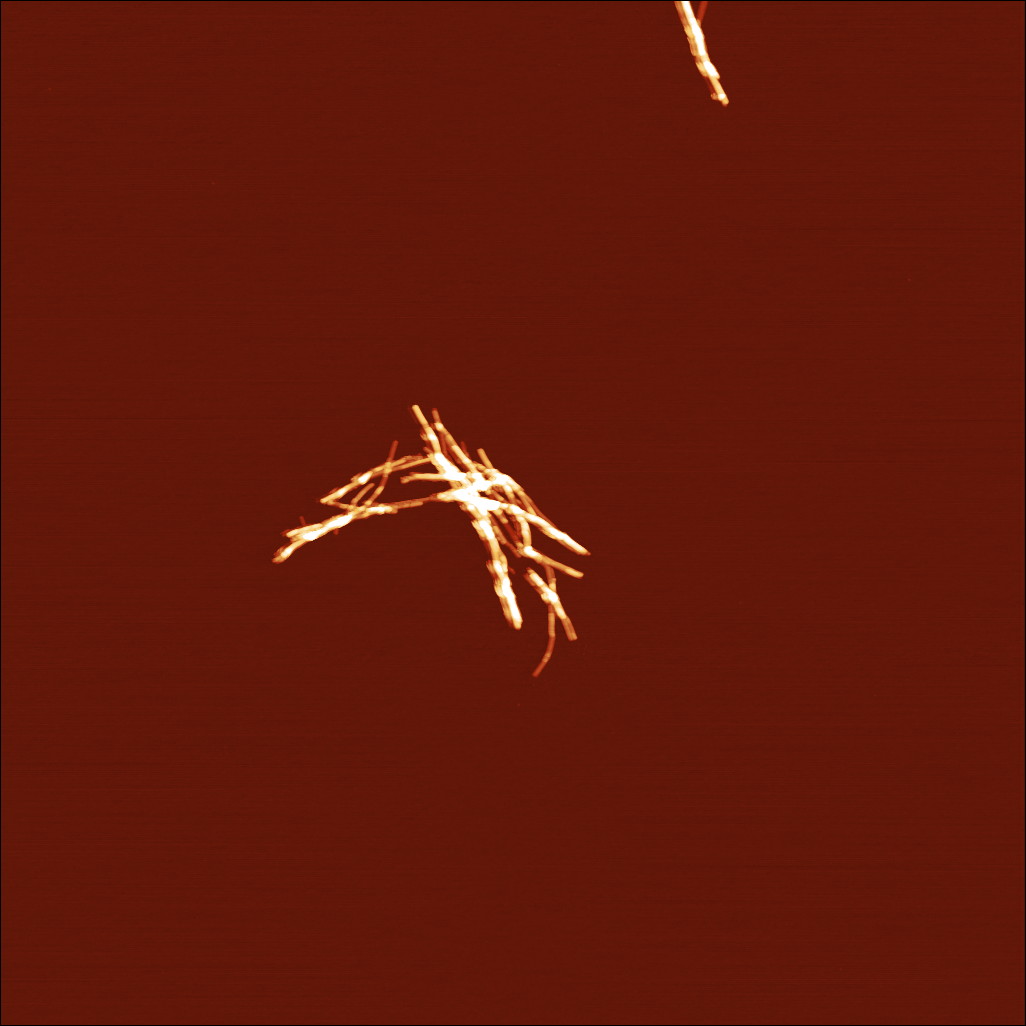

Supplement: Supplemental Information 5 [file peerj-09-10918-s010.zip › Normal2.tiff]

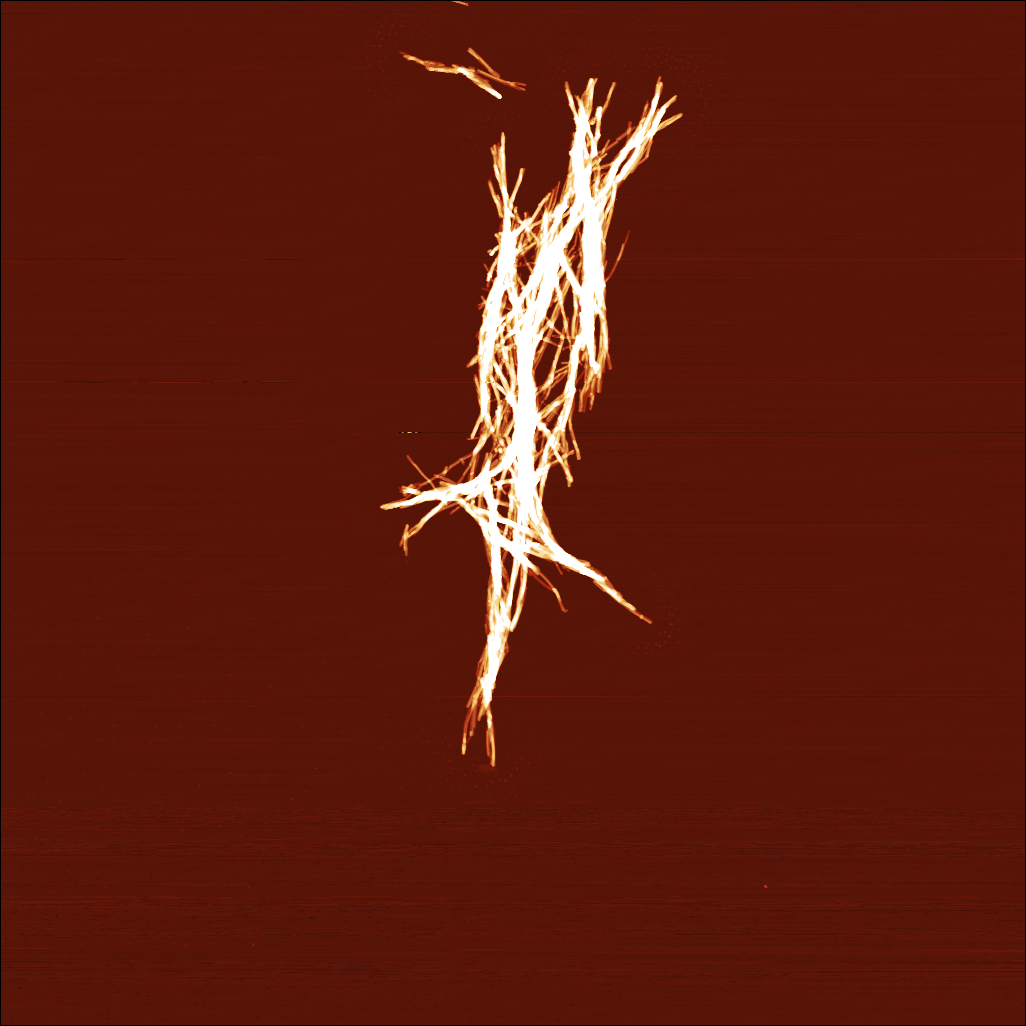

Supplement: Supplemental Information 5 [file peerj-09-10918-s010.zip › Normal3.tiff]

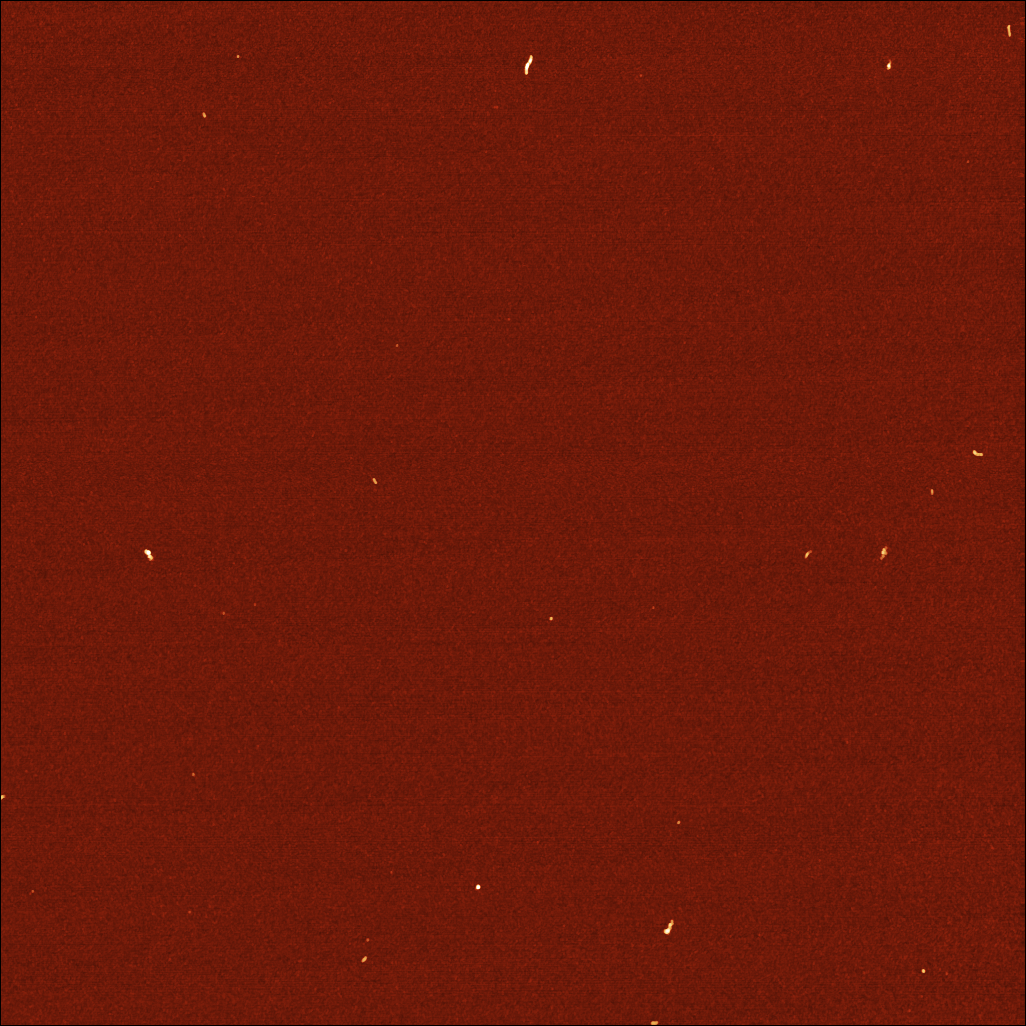

Supplement: Supplemental Information 5 [file peerj-09-10918-s010.zip › NormalIntermediate1.tiff]

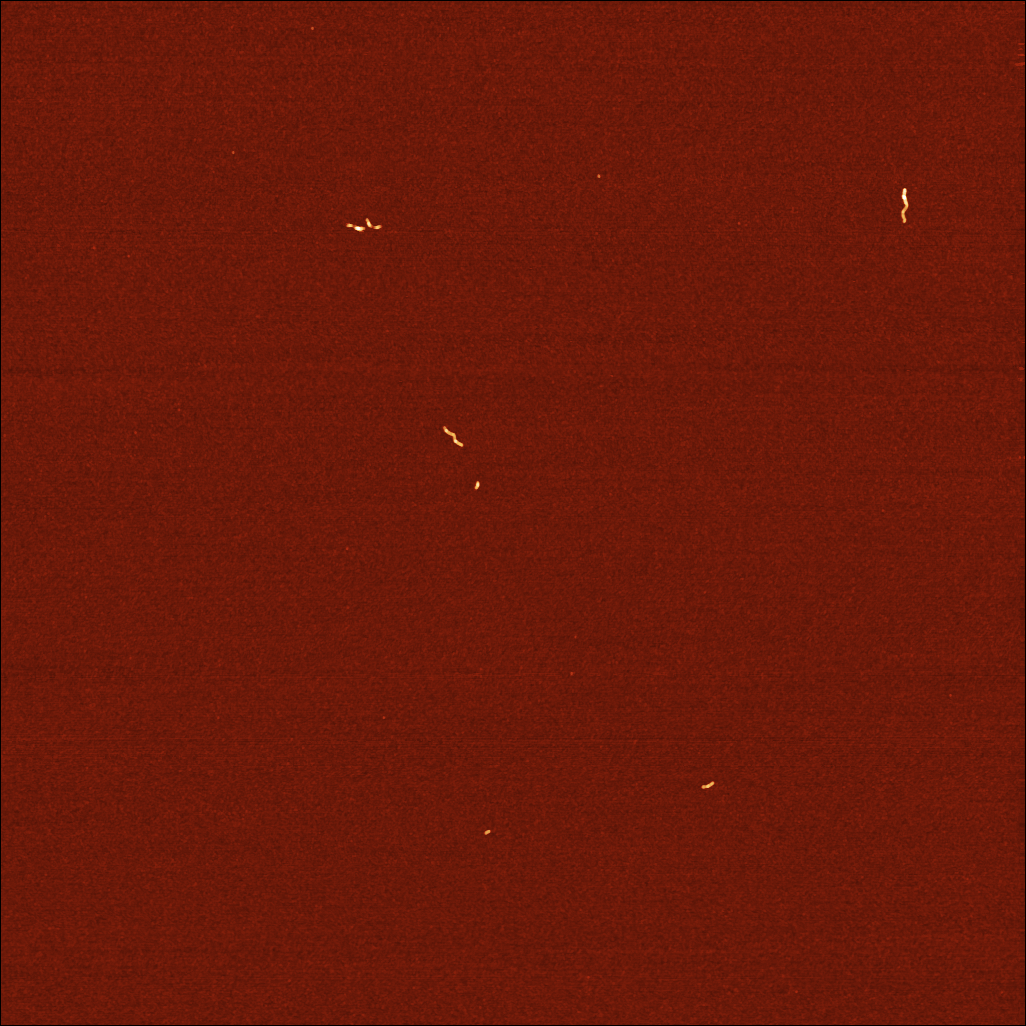

Supplement: Supplemental Information 5 [file peerj-09-10918-s010.zip › NormalIntermediate2.tiff]

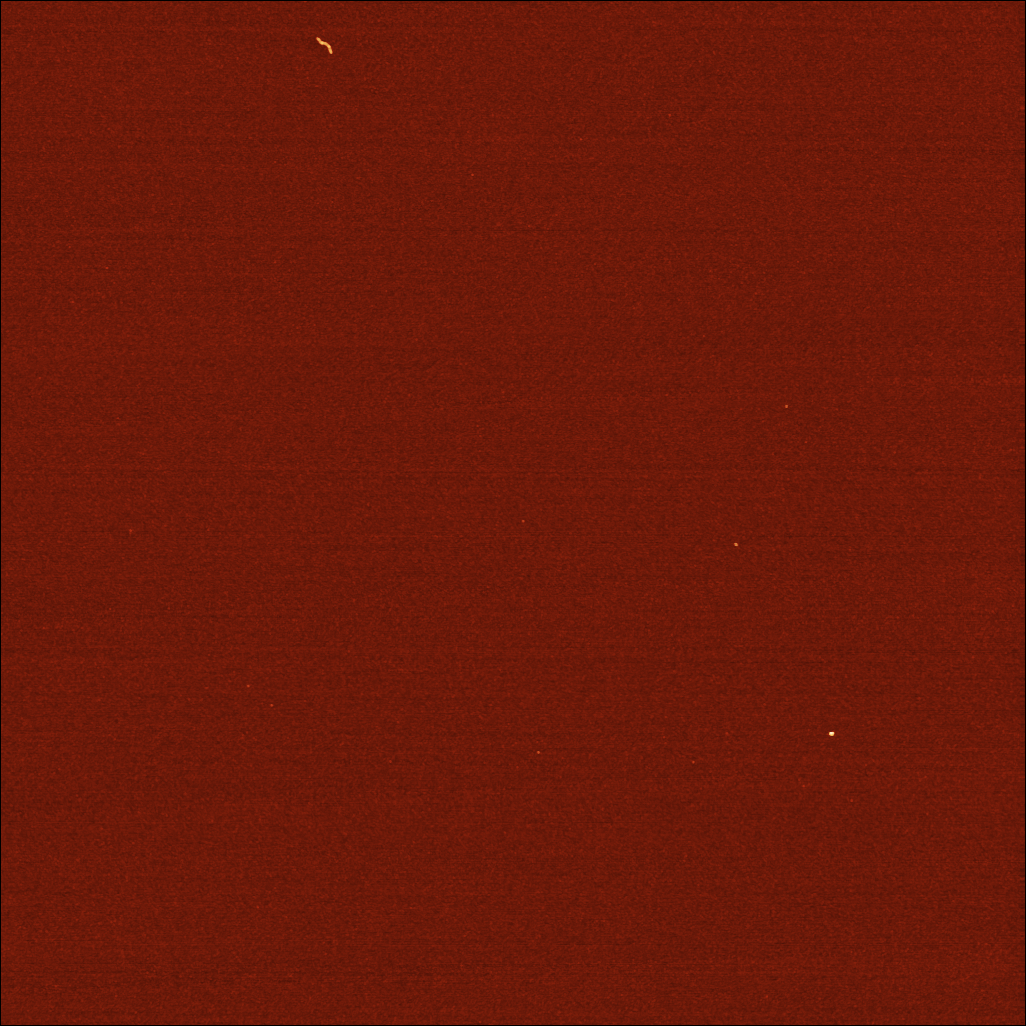

Supplement: Supplemental Information 5 [file peerj-09-10918-s010.zip › NormalIntermediate3.tiff]
